# A novel optical biosensor for the early diagnosis of sepsis and severe Covid-19: the PROUD study

**DOI:** 10.1186/s12879-020-05607-1

**Published:** 2020-11-19

**Authors:** Sarantia Doulou, Konstantinos Leventogiannis, Maria Tsilika, Matthew Rodencal, Konstantina Katrini, Nikolaos Antonakos, Miltiades Kyprianou, Emmanouil Karofylakis, Athanassios Karageorgos, Panagiotis Koufargyris, Gennaios Christopoulos, George Kassianidis, Kimon Stamatelopoulos, Robert Newberry, Evangelos J. Giamarellos-Bourboulis

**Affiliations:** 1 4th Department of Internal Medicine, National and Kapodistrian University of Athens, Medical School, 124 62 Athens, Greece; 2 Sanmina Corporation, 13000 S. Memorial Parkway, Huntsville, AL 35803 USA; 3 2nd Department of Critical Care Medicine, National and Kapodistrian University of Athens, Medical School, 124 62 Athens, Greece; 4 Intensive Care Unit, Korgialeneion Benakeion Athens General Hospital, 115 26 Athens, Greece; 5 Department of Therapeutics, National and Kapodistrian University of Athens, 115 28 Athens, Greece; 6 4th Department of Internal Medicine, ATTIKON University General Hospital, 1 Rimini Str, 12462 Athens, Greece

**Keywords:** Sepsis, Optical biosensor, Diagnosis, Severity, COVID-19, SARS-CoV-2

## Abstract

**Background:**

The accuracy of a new optical biosensor (OB) point-of-care device for the detection of severe infections is studied.

**Methods:**

The OB emits different wavelengths and outputs information associated with heart rate, pulse oximetry, levels of nitric oxide and kidney function. At the first phase, recordings were done every two hours for three consecutive days after hospital admission in 142 patients at high-risk for sepsis by placing the OB on the forefinger. At the second phase, single recordings were done in 54 patients with symptoms of viral infection; 38 were diagnosed with COVID-19.

**Results:**

At the first phase, the cutoff value of positive likelihood of 18 provided 100% specificity and 100% positive predictive value for the diagnosis of sepsis. These were 87.5 and 91.7% respectively at the second phase. OB diagnosed severe COVID-19 with 83.3% sensitivity and 87.5% negative predictive value.

**Conclusions:**

The studied OB seems valuable for the discrimination of infection severity.

## Background

Sepsis is the most common cause of death nowadays. A recent survey showed more than 48 million cases in 2017 worldwide, six million of which died [1]. The recent sepsis definition of sepsis as a life-threatening organ dysfunction associated with a dysregulated host response to an infection [2] allows for the severe infection by the novel SARS-CoV-2 (COVID-19) to be considered a case of sepsis since this is driven by a complex immune dysregulation of the host [3].

The early detection of sepsis is critical for management since favorable outcomes are associated with the start of treatment as fast as one hour [4–6]. Early diagnosis is, however, difficult to achieve in everyday clinical practice which is hampered by time delays for laboratory and radiological exams. Decision-making is often based on clinical judgment and on quick point-of-care testing. Pulse oximeter devices are often helpful to evaluate clinical severity but they miss specificity for a disease state. To achieve so, they need to be enriched with measurements indicating endothelial function like produced nitric oxide (NO).

In this study, we suggest that a novel optical biosensor (OB) point-of-care device that can integrate the readings of traditional pulse oximeters with additional wavelengths pulse photoplethysmography (PPG) techniques to provide information on endothelial function may rapidly evaluate infection severity. In the PROUD study, this OB is developed through two different phases each corresponding to different scenarios of infection severity. In the first phase, OB recordings were done at serial time intervals in patients at high-risk for sepsis in order to develop an algorithm that can perform efficient diagnosis. In the second phase, the algorithm was applied in a cohort of patients with viral infections in order to diagnose COVID-19 and subsequent severity.

## Methods

### First phase of PROUD study

PROUD (pulse PhotoplethysmogRaphy as an early tool for the diagnosis of sepsis thrOUgh a two-stage Development approach) was a clinical study that was conducted in four study sites (two departments of Internal Medicine and two Intensive Care Units) participating in the network of the Hellenic Sepsis Study Group (HSSG) (www.sepsis.gr). The study protocol (CIV-19-06-028824) was approved by the Ethics Committees of the participating study sites, by the National Ethics Committee of Greece (approval MD 3/19) and by the National Organization for Medicine of Greece. (approval MD 3/19; ClinicalTrials.gov; NCT04149132). The enrolment of patients took place between November 2019 and February 2020. Once the analysis of the data was available in March 2020 when the COVID-19 pandemic was prominent in Greece, it was considered appropriate to ask for an extension of the study to validate the results in patients with infection by SARS-CoV-2. This extension was approved by the National Organization for Medicines on March 30th 2020. Written informed consent was provided by the patients or by first-degree relatives in case of patients not able to consent. The patients analysed here have not been reported in any other submission by our group or anyone else.

Participants were adults of both genders at high risk for the development of sepsis. High risk for the development of sepsis was considered as the presence of any two of the following situations: a) any infection in a patient with total SOFA score less than 2; and b) patients with Charlson’s Comorbidity Index (CCI) more than 2 irrespective the reason of admission which is based on previous findings showing that CCI more than 2 is an independent predisposing factor for sepsis [7]. The rationale of the study design was to enrol infected patients without sepsis by the Sepsis-3 definitions but at high likelihood to aggravate their infection into sepsis due to their comorbidities.

Main exclusion criteria were age less than 18 years; any stage IV malignancy; do not resuscitate decision; active tuberculosis; and pregnancy or lactation. Enrolled patients were under follow-up by two groups of investigators, namely groups A and B, each being blind to the results of the other group. Group A investigators performed OB PPG recordings every two hours for three consecutive days. The OB was placed on the forefinger and each recording lasted for five minutes. The OB is a patented oximeter-like device that has been developed by Sanmina (Huntsville, AL) and works by measuring optical absorptions using reflectance techniques in five wavelengths i.e. 940 nm (IR), 660 nm (red color), 530 nm (green color), 465 nm (blue color) and 395 ± 10 nm (ultraviolet). The ratio of these wavelengths associates with vasoconstriction and vasodilation so as to provide information on the endothelial state. The recorded information was transmitted from the OB to a smartphone and from there to a cloud for data analysis. Group B investigators recorded the following information for three consecutive days: a) vital signs; b) type of infection; c) SOFA score; d) complete blood cell count and differential; e) biochemistry, PCT, CRP and blood gases; and f) microbiology. An amount of 3 ml of whole blood was sampled after venipuncture of one antecubital vein under aseptic conditions on the same days. Blood was immediately poured into one sterile and pyrogen-free tube that was placed on ice. The tube was transported immediately to the lab and centrifuged in 4 °C at 1500 g. NO was measured in the supernatant by the Griess reaction (Enzo Life Sciences Inc., Farmingdale, NY).

Based on the collected information, enrolled patients were classified into those who eventually developed sepsis during the 3-day intense follow-up and into those who did not develop sepsis. Classification into sepsis required both of the following [2]: a) presence of an infection; and b) increase of admission total SOFA score by at least two points.

The primary study endpoint of the first phase of PROUD was the accuracy of the OB for the diagnosis of sepsis at the timepoint of clinical diagnosis using the SOFA score. In order to achieve so, an algorithm that can provide the likelihood for sepsis at each time-point of sampling was developed. The working principle of the OB is emitting light into the local tissue using reflectance PPG techniques for 5 Wavelengths (940, 660, 530, 465, and 395 ± 10 nm). The OB device samples each wavelength absorbance approximately at 150 Hz and then recreates the arterial pulse pressure responses for each wavelength independently. Next, the device analyses the individual PPG wavelengths for each cardiac stroke synchronized to the systolic pulse pressure peak in order to calculate the a/c and d/c components encompassing the systolic and diastolic periods in the sampling window using the related volumetric changes of arterial blood at the specified wavelength dependent tissue depths. The information is subsequently used to calculate a series of parameters to compare the information from a blood analytical and vascular response point-of-view. For the blood analytical series of parameters, logarithmic (L) values are calculated for each wavelength. Subsequently R values are also calculated using the optical AC amplitude (pulsating PPG arterial signal) compared to the optical “DC” amplitude (non-pulsating arterial, venous, and tissue signals) using the eq. R = Iac (lambda 1) / Idc (lambda 1)/ Iac (lambda 2) / Idc (lambda 2). The risk of developing sepsis is aggregated by using a combination of calculations for the algorithm currently proposed including heart rate, relative vessel diameter, metabolites and a combination of L and R values related to NO and to oxygenated hemoglobin. This information generates the optical signatures via compiled Neural network (NN) training vectors. The output of this NN contains two algorithms; one on the confidence percentage for the positive likelihood for sepsis; and another on the negative likelihood for sepsis using 30-s sample windows of the optical biosensor data. Both algorithms have values ranging from 0 to 100. For the purpose of analysis, the means of all time readings of each patient were taken into use.

The correlation of the two algorithms was done by the Spearman’s rank of order. In order to evaluate the diagnostic performance of the algorithm, one Receiver Operator Characteristics (ROC) curve analysis was done using the Youden index to identify the best cut-off point for discrimination. Comparisons of quantitative data were done by the Student’s t-test for parametrical data and by the Mann-Whitney U test for non-parametrical data. Comparisons were done by the Fisher exact test for qualitative data. Odds ratios (ORs) and 95% confidence intervals (CIs) were calculated by Mantel-Haenszel statistics. Any *p* value less than 0.05 was considered significant.

The first phase of the study was powered for 139 patients. This was calculated in order to define a cut-off that can discriminate sepsis with 90% specificity with 90% power at the 5% level of significance. To adjust for possible missing values, 150 patients were enrolled.

### Second phase of the PROUD study

This phase started after the analysis of the data of the first phase. During this phase, participants were adults of both genders admitted at the emergencies with symptoms compatible with upper or lower respiratory tract infection. Main exclusion criteria were age less than 18 years; any stage IV malignancy; do not resuscitate decision; active tuberculosis; and pregnancy or lactation. All patients were subject to the following interventions: sampling of one nasopharyngeal swab; one single testing with the forefinger OB PPG point-of- care device for five minutes as described above; and one blood draw as described above. The recorded information was stored on a microSD card contained inside the OB device. The local time of synchronization and length of test was controlled by the smartphone, the microSD cards were individually retrieved, sterilized and the data was transferred to a storage device for data analysis. Each OB and smartphone was discarded following recording as safety precaution. Sampled swabs were subject to molecular detection of SARS-CoV-2. All patients with COVID-19 were subject to chest X-ray and/or chest computed tomography for the diagnosis of lower respiratory tract infection. Patients negative for SARS-CoV-2 were considered to have “flu-like” symptoms. NO was measured in the blood by the Griess reaction, as described above.

The diagnostic performance of the algorithm developed during the first phase was applied firstly to discriminate between COVID-19 and flu-like symptoms among all participants. It was then used to discriminate between severe and non-severe cases among all COVID-19 cases. Severe COVID-19 was diagnosed according to the WHO classification.

## Results

### The two phases of the PROUD study

**Fig. 1:**
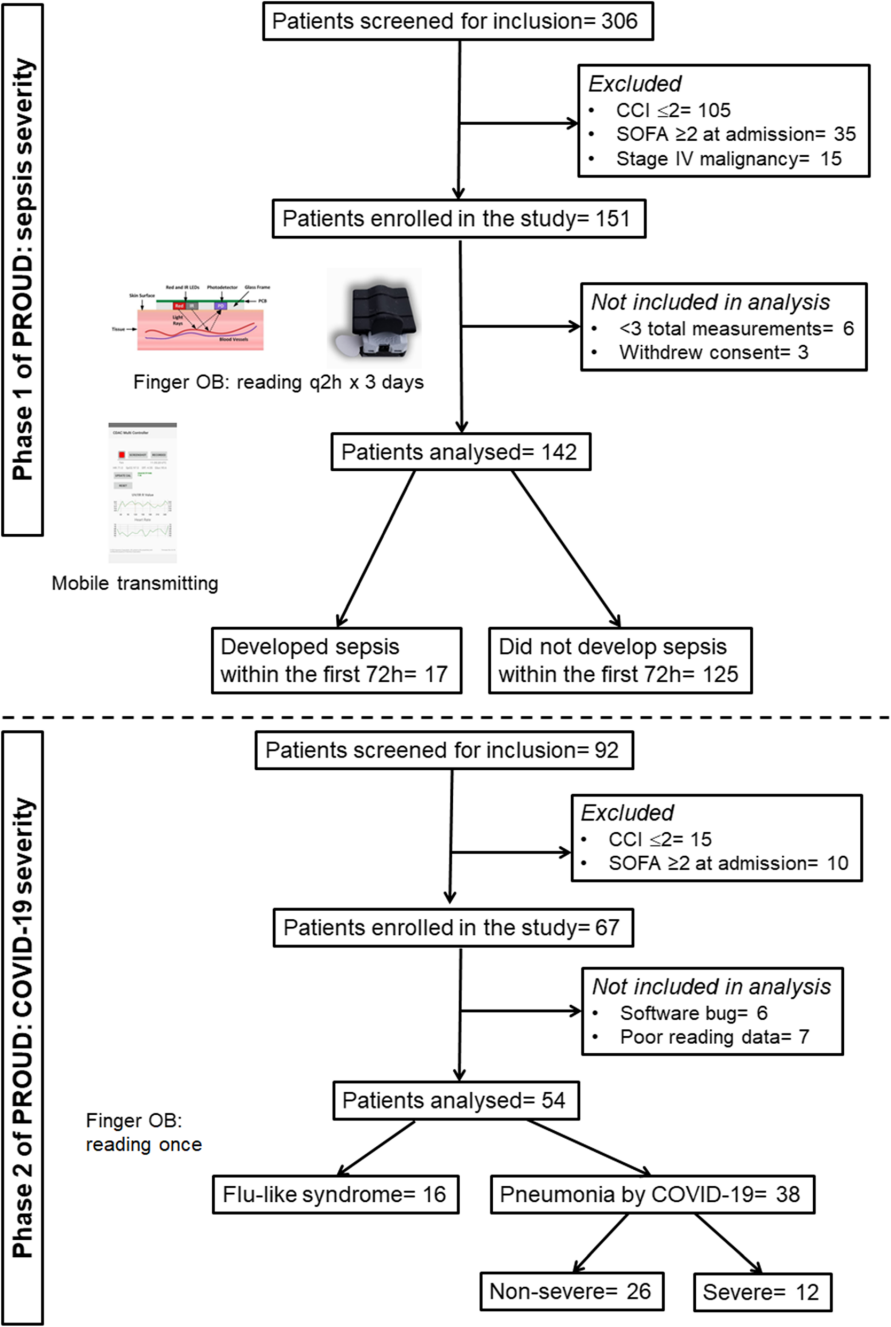
Study flow-chart. Abbreviations: CCI: Charlson’s comorbidity index; OB: optical biosensor; q2h: every two hours; SOFA: sequential organ failure assessment

The PROUD study had two phases: one first phase that took place between November 2019 and February 2020 trying to develop the OB PPG point-of-care device as a test for the discrimination of sepsis and organ dysfunction; and one second phase that took place between April 2020 and May 2020 and investigated the ability of the developed algorithm for the detection of severity among patients with pneumonia by SARS-CoV-2. The study flow chart is shown in Fig. 1.

### Development of an algorithm for sepsis diagnosis

**Fig. 2:**
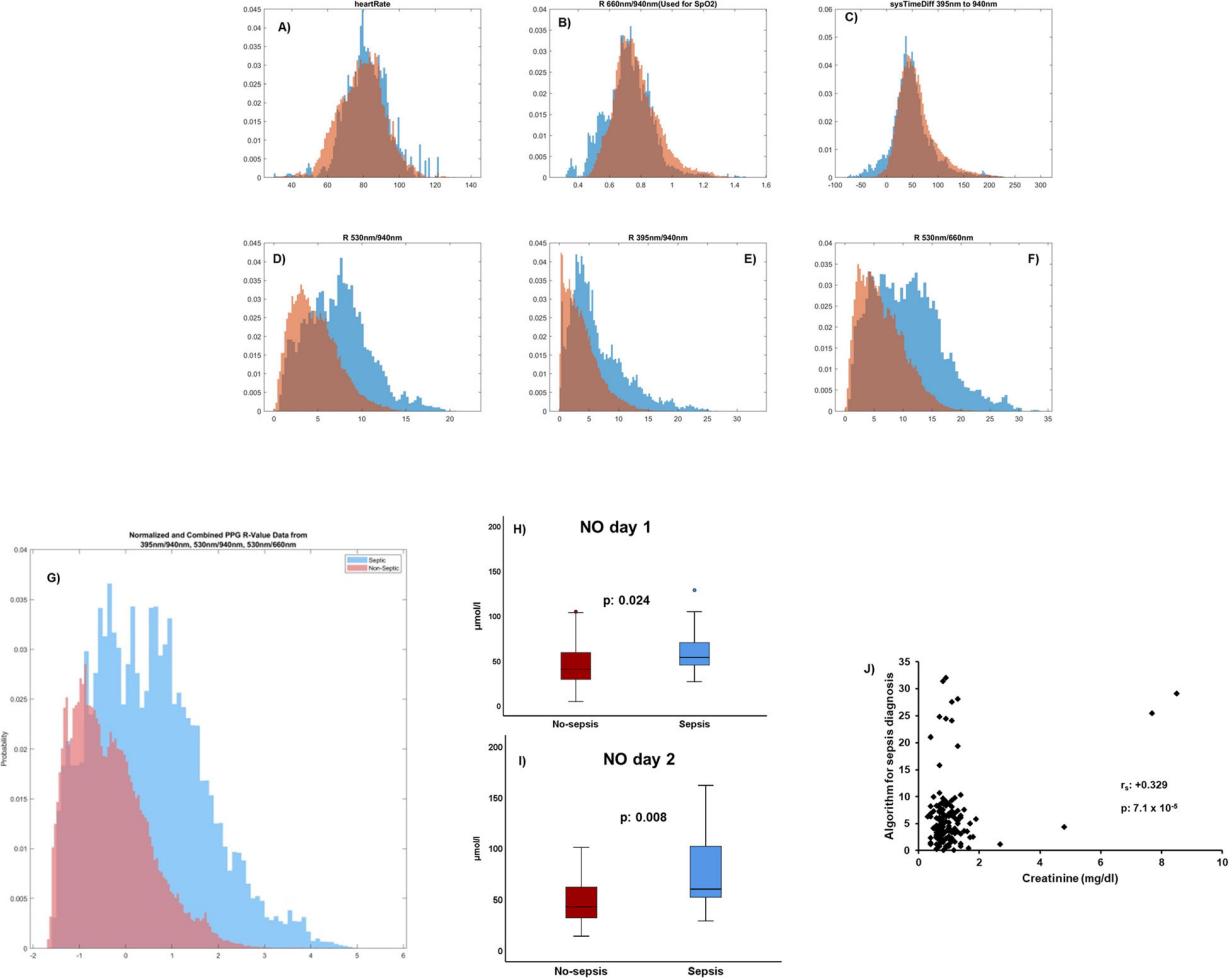
Basic elements of the sepsis classification tool. Panels A to G are histograms comparing the absorption rates of the PPG optical biosensor (OB) between patients with sepsis (in blue) and not in sepsis (in dark red). **a**) Heart Rate. **b**) R 660/940 nm: absorption of oxygenated versus de-oxygenated hemoglobin. **c**) sysTimediff (395 to 940 nm): the difference in time between the systolic points in 395 to 940 nm in millisecond providing an approximation of the vessel diameter. **d**) R 530/940 nm: information on kidney function **e**) R 395/940 nm: levels of nitric oxide (NO). **f**) R 530/660 nm: ratio expressing poor oxygen absorption due to inflammatory interferences. **g**) Integration of absorption ratios 530/940 nm, 395/940 nm and 530/660 nm for sepsis classification. **h**) NO levels in the blood measured on day 1 by the Griess reaction. Circles denote outliers. The provided *p*-value refers to the comparison between non-sepsis and sepsis by the Mann-Whitney U test. **i**) NO levels in the blood measured on day 2 by the Griess reaction. Circles denote outliers. The provided p-value refers to the comparison between non-sepsis and sepsis by the Mann-Whitney U test. **j**) Correlation between the calculated algorithm of the OB and serum creatinine. The Spearman’s co-efficient of correlation (r_s_) and the respective p-value are provided

At the first phase of the study 142 patients were enrolled; 17 developed sepsis during the 3-day follow-up (Table 1). The developed neural network was fed with six different types of information: heart rate; the absorption ratio of 660/940 nm of oxygenated versus de-oxygenated haemoglobin; the difference in time between the systolic points in 395 to 940 nm providing an approximation of the vessel diameter; the absorption ratio 530/940 nm reflecting to creatinine levels; the absorption ratio 395/940 nm reflecting to the NO levels; and the absorption ratio 530/660 nm expressing poor oxygen absorption due to inflammatory interferences. The comparative histograms for these ratios between non-sepsis and sepsis patients provided clear discrimination between the two states (Fig. 2a to g). Measured NO in the blood of the first two days was significantly higher in sepsis patients and corroborated the findings from the 395/940 nm absorption ratio (Fig. 2h and i). Furthermore, a positive correlation between the algorithm of the OB and serum creatinine was found verifying that the OB algorithm provides information of the renal function (Fig. 2j).

**Table 1:** Baseline characteristics of enrolled patients divided into those who developed sepsis and into those who did not develop sepsis

	Sepsis (***n*** = 17)	Non-sepsis (***n*** = 125)	***p***-value
Age years, mean (± SD)	73.4 ± 13.4	73.5 ± 14.7	0.960
Male gender, n (%)	9 (52.9)	5 (45.6)	0.612
Admission APACHE II, mean (± SD)	10.88 ± 3.72	7.73 ± 2.90	< 0.0001
Admission SOFA score, mean (± SD)	2.59 ± 2.15	1.08 ± 1.49	< 0.0001
Time of sepsis onset (min), median (IQR)	1080 (1760)	NA	
CCI, mean ± SD	5.24 ± 2.53	4.58 ± 2.27	0.270
Comorbidities, n (%)			
Type 2 diabetes mellitus	7 (41.2)	36 (28.8)	0.398
Chronic obstructive pulmonary disease	3 (17.6)	8 (6.4)	0.128
Chronic heart failure	7 (41.2)	21 (16.8)	0.045
Chronic renal disease	3 (17.6)	11 (8.8)	0.377
Stroke	1 (5.9)	28 (22.4)	0.196
Dementia	2 (11.8)	13 (10.4)	1.00
Coronary heart disease	2 (11.8)	19 (15.2)	1.00
Atrial fibrillation	6 (35.3)	23 (18.4)	0.116
Depression / psychosis	0 (0)	14 (11.2)	0.219
Intake of antimicrobials the last 3 months	5 (29.4)	28 (22.4)	0.545
Underlying infections, n (%)			
Respiratory tract infections	6 (35.3)	31 (24.8)	0.382
Urinary tract infection	2 (11.8)	13 (10.4)	1.00
Intra-abdominal infection	5 (29.4)	10 (8)	0.019
ABSSSI	2 (11.8)	6 (4.8)	0.245
Bacteremia	1 (5.9)	1 (0.8)	0.226
Other	1 (5.9)	7 (5.6)	0.477
White blood cells (/mm^3^, mean ± SD)	9852 ± 5209	9550 ± 5868	0.841
Platelets (× 10^3^/mm^3^, mean ± SD)	221 ± 100	247 ± 79	0.291
INR (mean ± SD)	1.10 ± 0.17	1.15 ± 0.43	0.736
Creatinine (mg/dl, mean ± SD)	1.88 ± 2.44	0.94 ± 0.49	0.001
CRP (mg/l, median-IQR)	62.2 (74.1)	18.3 (83.8)	0.140
PCT ng/ml, median (IQR)	0.26 (0.64)	0.11 (0.22)	0.025

*Abbreviations*: *ABSSSI* Acute bacterial skin and skin structure infection, *APACHE* Acute physiology and chronic health evaluation, *CCI* Charlson’s comorbidity index, *CRP* C-reactive protein; INR: International normalized ratio, *IQR* inter-quartile range, *PCT* procalcitonin, *SOFA* sequential organ failure assessment

**Fig. 3:**
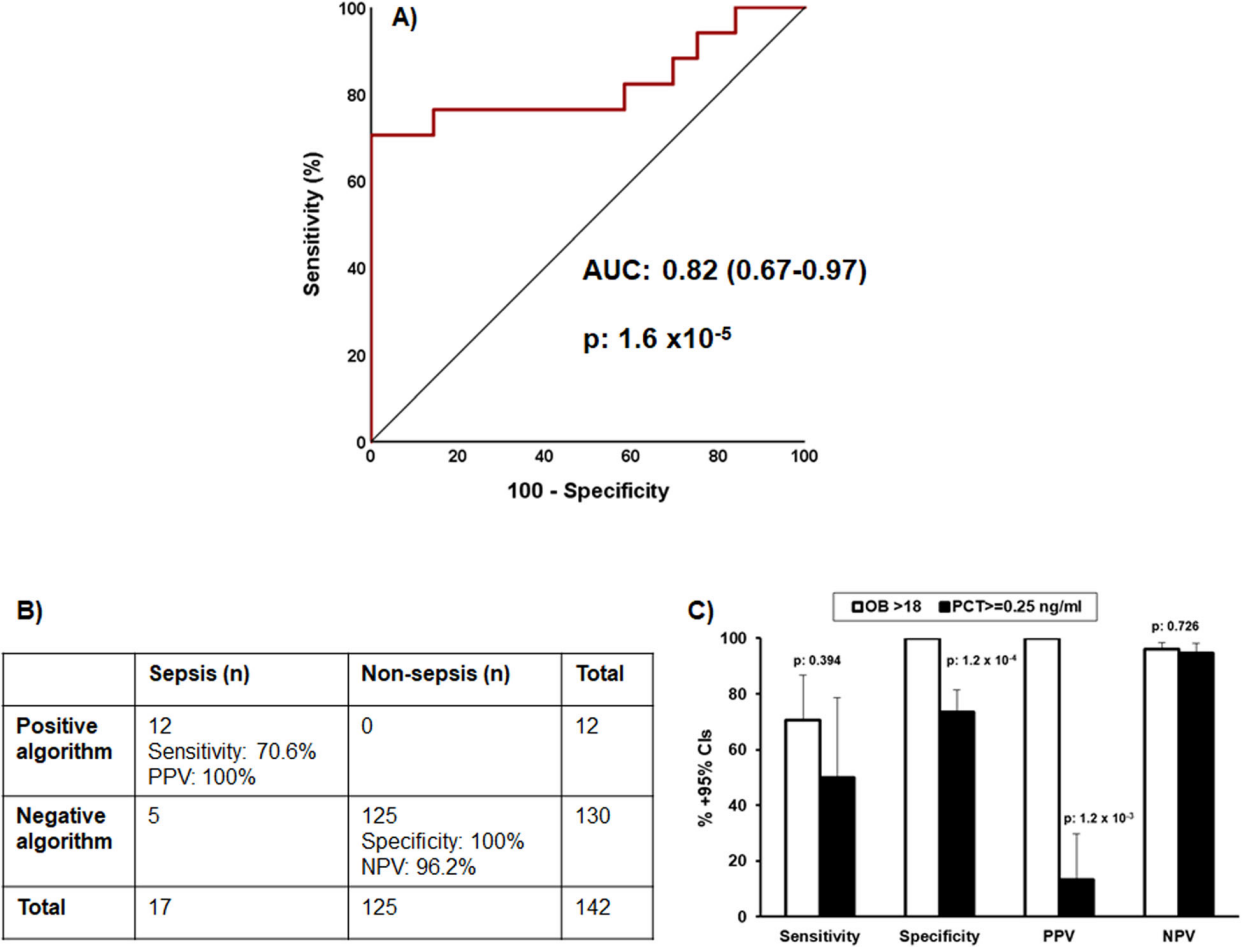
Diagnostic performance of the calculated algorithm for sepsis. **a**) ROC curve of the algorithm for the diagnosis of sepsis. The area under the curve (AUC), the confidence intervals and the *p* value of significance are provided. **b**) Sensitivity, specificity, positive predictive value (PPV) and negative predictive value (NPV) of an algorithm value greater than 18 for the diagnosis of sepsis. **c**) Comparative diagnostic performance of an OB algorithm value greater than 18 and of procalcitonin (PCT) greater than 0.25 ng/ml for the diagnostic of sepsis. The *p*-values of the indicated comparisons are provided. CI: confidence interval

The two algorithms of the positive and negative likelihood for sepsis had an almost absolute correlation (r_s_: − 0.972; p: 1.8 × 10^− 80^) showing that practically the one was the inverse of the other. As such, further analysis was done only by using the algorithm for the positive likelihood. ROC curve analysis identified a cut-off greater than 18 that could provide diagnosis of sepsis with 70.6% sensitivity, 100% specificity, 100% positive predictive value and 93.2% negative predictive value (Fig. 3a and b). The specificity and the positive predictive value of the OB at the 18 cut-off was significantly greater than that of the inflammatory biomarker procalcitonin (PCT) (Fig. 3c). However, among the five patients who developed sepsis and who were scoring false-negative by the OB, PCT was greater than 0.25 ng/ml in three patients. In this case, the integration of PCT to the OB prediction increased the sensitivity for the diagnosis of sepsis to 88.2%.

### Scenario of severity detection in patients with COVID-19

The second phase of the PROUD study had two endpoints: a) to investigate the developed algorithm for the positive likelihood among patients with pneumonia by SARS-CoV-2 compared to patients with flu-like symptoms; and b) to study if this algorithm may predict severe COVID-19 (Table 2).

**Table 2:** Baseline characteristics of enrolled patients with COVID-19 divided into severe and non-severe cases

	Severe (n = 12)	Non-severe (***n*** = 26)	***p***-value
Age (years, mean ± SD)	68.1 ± 11.2	63.2 ± 18.5	0.405
Male gender, n (%)	8 (33.3)	16 (66.7)	1.00
APACHE II score (mean ± SD)	10.1 ± 3.6	8.2 ± 5.8	0.297
SOFA score (mean ± SD)	3.6 ± 1.4	1.5 ± 1.8	0.001
CCI score (mean ± SD)	3.2 ± 2	3.4 ± 2.7	0.804
Comorbidities, n (%)			
Type 2 diabetes mellitus	4 (44.4)	5 (55.6)	0.423
Chronic heart failure	0	4 (100)	0.287
Chronic renal disease	0	5 (100)	0.158
Chronic obstructive pulmonary disease	2 (28.6)	5 (71.4)	1.00
Solid malignancy	2 (40)	3 (60)	0.643
Chemotherapy	2 (40)	3 (60)	0.643
Dementia	1 (25)	3 (75)	1.00
Atrial fibrillation	0	3 (100)	0.538
Residency in long-term healthcare facility	0	3 (100)	0.538
Previous intake of antibiotics	2 (12.5)	14 (87.5)	0.040
White blood cells (/mm^3^, mean ± SD)	9334 ± 2498	6194 ± 3578	0.010
Platelets (× 10^3^ /mm^3^, mean ± SD)	252 ± 116	282 ± 140	0.530
INR (mean ± SD)	1.1 ± 0.1	1.1 ± 0.3	0.776
Creatinine (mg/dl, mean ± SD)	1.0 ± 0.9	1.8 ± 1.9	0.216
CRP (mg/l, median ± IQR)	63.8 ± 133.5	26.5 ± 41.6	0.006
PCT (ng/ml, median ± IQR)	0.1 ± 0.7	0.1 ± 0.1	0.814
pO_2_/FiO_2_ (mean ± SD)	211.7 ± 85.1	389.3 ± 98.6	< 0.001

*Abbreviations*: *APACHE* Acute physiology and chronic health evaluation, *CCI* Charlson’s comorbidity index, *CRP* C-reactive protein, *FiO*_*2*_ fraction of inspired oxygen, *INR* International normalized ratio, *IQR* inter-quartile range, *PCT* procalcitonin, *pO*_*2*_ partial oxygen pressure, *SOFA* sequential organ failure assessment

**Fig. 4:**
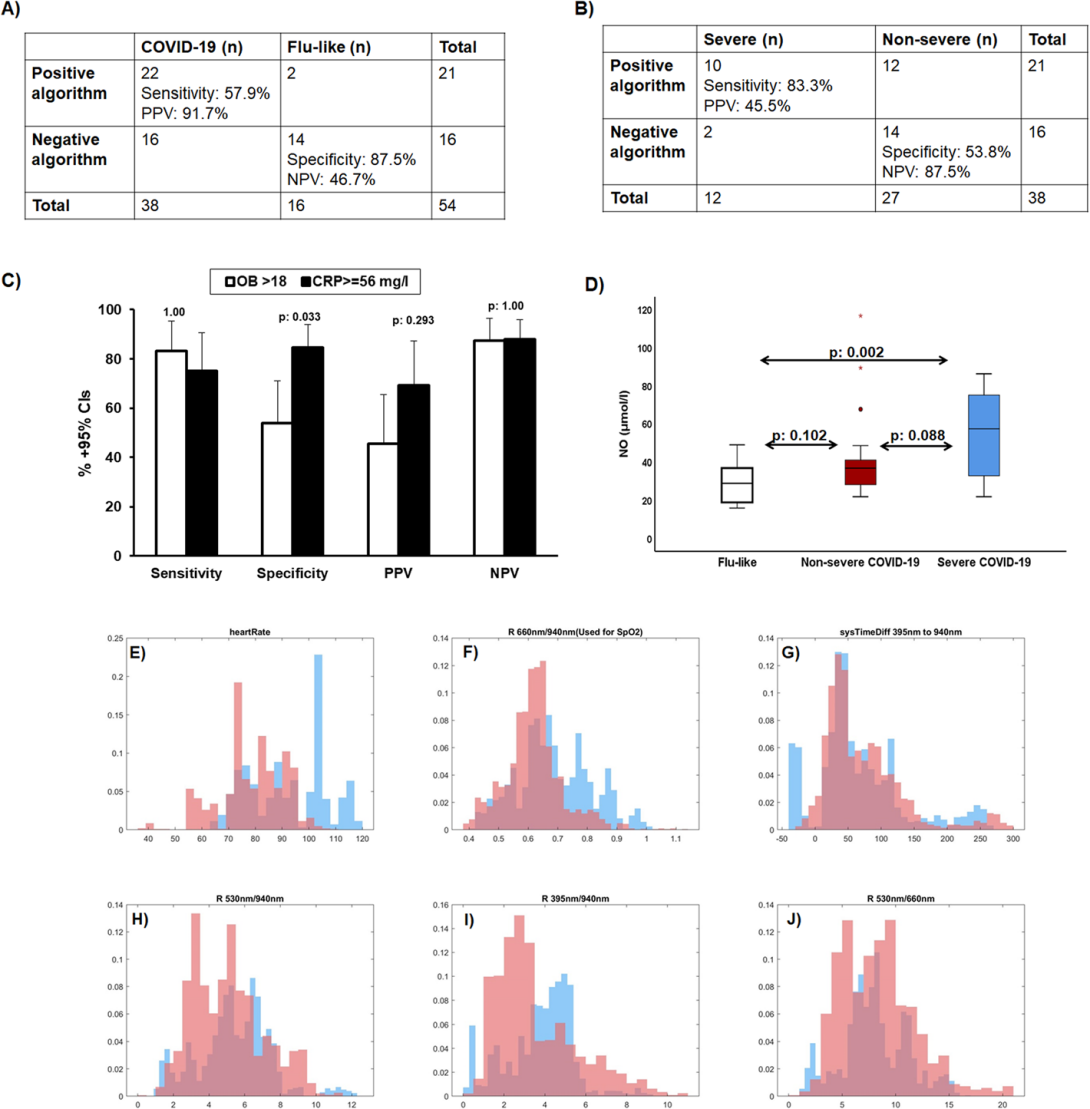
Validation of the diagnostic algorithm in COVID-19. **a**) Sensitivity, specificity, positive predictive value (PPV) and negative predictive value (NPV) of an OB algorithm value greater than 18 for the diagnosis of COVID-19. **b**) Sensitivity, specificity, positive predictive value (PPV) and negative predictive value (NPV) of an algorithm value greater than 18 for the diagnosis of severe COVID-19. **c**) Comparative diagnostic performance of an OB algorithm value greater than 18 and of C-reactive protein (CRP) greater or equal to 56 mg/l for the diagnosis of severe COVID-19. The 56 mg/l of CRP was defined after co-ordinate point analysis of the ROC curve. The p-values of the indicated comparisons are provided. CI: confidence interval. **d**) NO levels in the blood measured by the Griess reaction. Circles denote outliers and asterisks denote extremes. The p-values of the indicated comparisons by the Mann-Whitney U test are shown. Panels E to J are histograms comparing the absorption rates of the PPG optical biosensor between patients with severe COVID-19 (in blue) and non-severe COVID-19 (in dark red). **d**) Heart Rate. **e**) R 660/940 nm: absorption of oxygenated versus de-oxygenated hemoglobin. **f**) sysTimediff (395 to 940 nm): the difference in time between the systolic points in 395 to 940 nm in millisecond providing an approximation of the vessel diameter. **g**) R 530/940 nm: information on kidney function. **h**) R 395/940 nm: levels of nitric oxide (NO). **i**) R 530/660 nm: ratio expressing poor oxygen absorption due to inflammatory interferences

At the cut-off value of 18 of the algorithm for the positive likelihood, COVID-19 was diagnosed with 57.9% sensitivity, 87.5% specificity, 91.7% positive predictive value and 46.7% negative predictive value (Fig. 4a) that were similar to the diagnostic performance for sepsis (OR 9.62; 95% CIs 1.91–48.42; p: 0.006). This cut-off could discriminate severe COVID-19 with 83.8% sensitivity and 87.5% negative predictive value (Fig. 4b) (OR 5.83; 95% CIs 1.06–32.02; p: 0.040). All these severe patients were admitted in an intensive care unit under mechanical ventilation. At that OB cut-off the diagnostic performance to discriminate severe from non-severe infection had similar sensitivity, positive predictive value and negative predictive value to C-reactive protein (CRP), but lower specificity than CRP (Fig. 4c). When circulating NO was measured in patients with flu-like syndrome, in patients with non-severe COVID-19 and in patients with severe COVID-19, it was found that NO was significantly greater in severe COVID-19 (Fig. 4d). Following the measurement of circulating NO, the histograms of the absorption ratios were analysed to identify which the component between the six measured variables that impacted more on the discrimination between non-severe and severe COVID-19 was (Fig. 4e to j). The absorption ratio 395/940 nm reflecting the NO levels (Fig. 4i) had most of the impact.

## Discussion

In this study we used a two-step approach for the development of an OB point-of-care device that is based on PPG for the diagnosis of severe infections. The OB integrated information from heart rate, pulse oximetry, kidney function, NO levels, vascular diameter and presence of inflammation and provided at the first phase diagnosis of sepsis with 100% specificity and 100% positive predictive value so as to perform better than biomarkers. The use of PCT may assist to increase the sensitivity for the diagnosis of sepsis. The merit of this first phase is the exhaustive study design necessitating recording every two hours for three consecutive days so as to coincide recording with sepsis diagnosis. When the COVID-19 pandemic arrived, we asked if this OB could assist in a different emerging scenario for the diagnosis of infection by SARS-CoV-2 and for the detection of severity among patients admitted with symptoms compatible for viral infections. It needs to be outscored that contrary to the first phase that involved serial recordings, the second phase contained one single recording that was interpreted based on the set-up of the OB algorithm from the first phase. At this second phase, one single recording could provide accurate assessment of COVID-19 severity. There is no doubt that one OB reading cannot diagnose the etiology of one viral infection. However, in the light of the current pandemic where every admission at the emergencies bears the suspicion of COVID-19 and where final molecular diagnosis delays by several hours, the availability of a tool that can early trace severity and prompt early action becomes a valuable assistant. OB was similar to the CRP for the discrimination of patients with severe infection. This last observation was of major importance since it discloses the financial benefit for the health system introduced with the new OB: a) the OB is reusable so as to save money from biomarker measurements; and b) it provides a diagnostic output much faster that the lab analysis requested for CRP.

Pulse oximetry is a technique that is used for the monitoring of the respiratory function and of the heart rate which, however, lacks specificity for any disease. The integration of information from vascular damage, NO levels and kidney function in the new OB transforms pulse oximetry into a diagnostic panel for severe infections. Vascular dysfunction associated with failure of the endothelial function is the main culprit for tissue hypo-perfusion in sepsis and over-production of NO plays a major role in tissue vasodilation [8, 9]. Although endothelial damage is not a prominent feature of viral infections, our data indicate that COVID-19 complicated by lower respiratory tract infection leads to profound endothelial damage which is traced by the OB PPG point-of-care device. Indeed, post-mortem lung histology of 21 patients with severe COVID-19 revealed significant vascular damage dominated by diffuse exudation in the alveoli, vascular microthrombi and vasculitis [10–12].

The main study limitations are: the limited number of patients who eventually developed sepsis during the first phase; and single recordings during the second phase. The interpretation of the OB PPG device should be done with caution when patients suffer from chronic disorders that may interfere with measurements like arrhythmias, chronic liver failure and chronic renal disease.

## Conclusions

The new OB integrating information of respiratory, renal and endothelial function is a new diagnostic tool for the assessment of infection severity. The presented data generate hopes that this OB may become a valuable tool for two main reasons: a) the rapid detection of sepsis as compared to other markers which may delay early diagnosis and treatment; and b) the feasibility of testing when the infectious environment is highly contagious, as is the case with the COVID-19 pandemic and where the dressing of the physician limits mobility and traditional diagnostic work-up. However, testing in larger cohorts is still needed.

## Data Availability

Data are available from the corresponding author upon reasonable request.

## Abbreviations

CCI: Charlson’s comorbidity index
CI: confidence interval
COVID-19: coronavirus disease 2019
CRP: C-reactive protein
NO: nitric oxide
NPV: negative predictive value
OB: optical biosensor
OR: odds ratio
PCT: procalcitonin
PPG: pulse photoplythesmography
PPV: positive predictive value
ROC: receiver operator characteristics
SARS-CoV-2: severe acute respiratory syndrome coronavirus 2
SOFA: sequential organ failure assessment
